# The truth project- paper two- using staff training and consultation to inculcate a testimonial sensibility in non-specialist staff teams working with survivors of child sexual abuse

**DOI:** 10.3389/fpsyt.2023.1177622

**Published:** 2023-07-04

**Authors:** Claire Barker, Daniel Taggart, Marta Gonzalez, Sally Quail, Rebekah Eglinton, Stephanie Ford, William Tantam

**Affiliations:** ^1^IICSA, London, United Kingdom; ^2^School of Health and Social Care, University of Essex, Colchester, United Kingdom; ^3^Government of the United Kingdom, London, United Kingdom; ^4^School of Health and Social Care, University of Bristol, Bristol, United Kingdom

**Keywords:** epistemic justice, child sexual abuse, staff training, Public Inquiry, trauma informed approach

## Abstract

This paper explores how trauma informed training and consultation for non-specialist staff at the Independent Inquiry into Child Sexual Abuse in England and Wales enabled them to work with survivors of non-recent child sexual abuse in the Truth Project and other areas of the Inquiry. The paper draws on data gathered from 32 semi-structured interviews with a range of Inquiry staff, including civil servants, legal professionals, senior operational managers, and researchers. The interview questions mapped on to the trauma informed principles embedded in the Inquiry and considered the efficacy and implementation of this training for engaging with survivors’ voices, working with challenging testimonies and materials, and contributing to epistemic change. Findings included all staff having an awareness of what it meant to be trauma informed in an Inquiry context, talking about the principles in terms of value-based positions. Staff described an awareness of needing to attend to the idiosyncratic experiences of the individual survivor, and there was recognition that previous damage to survivor trust, through institutional failure, meant that demonstrating trustworthiness was a central task. Staff talked about the impacts of participation on some survivors, and the impacts it had on them to be exposed to trauma-related materials. There was acknowledgment of the limitations of the trauma informed approach but also recognition of the wider applications of this learning for other areas of their personal and professional lives. There is some support for the therapeutic culture developed at the Inquiry leading to what Fricker refers to as a testimonial sensibility, a quality of listening necessary for the establishment of epistemic justice. The discussion focuses on how this way of working can be applied to other public service settings and how epistemic justice concepts can be included in more traditional trauma informed care models to encourage an ethic of listening that has political and social, in addition to therapeutic, outcomes.

## Introduction

This article aims to evaluate the impacts of training staff in trauma informed approaches through an analysis of staff experiences at the Independent Inquiry into Child Sexual Abuse (‘IICSA’ or ‘the Inquiry’ hereafter). We consider staff perspectives on the Trauma Informed Approach (TIA) training and consultation they received, the impacts it had on their engagement with survivor and survivor testimonies, and the individual professional and personal reverberations such training engendered. We analyze their experiences via the lens of epistemic justice, using aspects of Fricker’s work on testimonial justice to consider the tensions staff faced between offering a survivor-centered service while working in a civil service role (1). One of the key findings concerns how staff were able to bring aspects of their own values and life experience into their work at IICSA and a reciprocal shift in their world view around child sexual abuse (CSA).

IICSA was established as an Inquiry in 2015 to investigate institutional failures to protect children from sexual abuse in England and Wales. It also made meaningful recommendations in order to contribute to institutional change. From 2016 to 2021, over 6,000 adult victims and survivors participated in Truth Project sessions, in which they could share experiences about child sexual abuse without prompt and in their own words. The IICSA’s Victims and Survivors Consultative Panel (VSCP), a group of CSA survivors who have expertise in the field, co-designed the Truth Project and contributed to ensuring that victim and survivor voices were represented throughout the process of receiving and processing these experiences.

The aims of this study were to understand the extent to which staff who may or may not have previously engaged with work relating to survivors were able to inculcate a trauma informed, testimonial sensibility in relation to survivor experience and testimonies. As the largest Inquiry of its kind to date, IICSA offers a hitherto unparalleled context within which to reflect on the ability of non-specialist staff-wide training programs to meaningfully equip individuals to deliver a trauma informed service in an Inquiry setting.

This article begins with a consideration of the meaning and implementation of trauma informed approaches in order to situate the particular training and practices of IICSA staff. It goes on to consider Fricker’s work in relation to shifting staff orientations to epistemologies and justice, and the concomitant changes to survivor voice and testimones (1). It moves on to detail the research methodologies of qualitative, semi-structured interviewing, and engages with the qualitative interview data provided by 32 research participants and considers the key findings relating to application of trauma informed models, trust, individual experiences, empowerment, and personal growth. Finally it offers suggestions relating to policy and practice outside of specific therapeutic contexts.

### Staff and trauma informed approaches (TIAs)

Trauma informed services are based on collaborative relationships between service providers and survivors (2). This is because the relational context in which abuse occurs means that any attempts to heal trauma requires forms of relating that are different from abusive dynamics. Trauma survivors often struggle to engage with services that replicate features of controlling and coercive relationships that mirror abusive relationships in childhood (3). Staff training and development is therefore a central feature of the organizational change process of embedding TIAs in frontline services.

Overall, TIAs provide inconsistent evidence in support of treating psychological outcomes (4). There is some evidence that they are effective in reducing Post-Traumatic Stress Disorder (PTSD) and anxiety symptoms but more research is needed to identify specific mechanisms of change, given the heterogenous nature of TIAs. While the majority of TIA interventions are delivered by clinicians with previous training in mental health, there is some support for non-specialist lay staff being able to effectively utilize TIAs if properly trained and supported (5, 6).

TIA staff training typically includes consideration of staff wellbeing and associated constructs such as secondary and vicarious traumatisation (7). The evidence about its effectiveness in this respect is mixed, with one study finding vicarious trauma symptoms increased following a training intervention (8). The authors of this study concluded that it was the increased awareness of the underlying trauma histories that drove service user’s presenting difficulties and the need for an attitude and behavioral shift from control to care in a youth justice setting, that may have led to the shift. It is therefore important to consider staff wellbeing in any evaluation of TIA training and implementation, as staff outcomes may be more nuanced than expected.

### Staff training and consultation at IICSA

IICSA undertook a particular form of TIA based on its status as a Public Inquiry. The Inquiry staff were multi-disciplinary with a majority being civil servants. Staff training and a psychological consultation service were central components of the TIA implementation. TIAs are an organizational level intervention that recognize the health and social impacts of traumatic stress and have an awareness of the ways that institutions may reenact traumatic dynamics when delivering services to victims and survivors (3). TIAs recognize the impacts of trauma, while also structuring the organization and the practices of staff to minimize the risks of retraumatization (9). Within IICSA, the TIA model was comprised of 5 key principles; (1) Recognizing that the experience of child sexual abuse is subjective and individuals should be respected; (2) Being aware that trust is not to be taken for granted, but fostered; (3) Empowering victims and survivors in their interactions with the Inquiry; (4) Prioritizing the safety and well-being of victims and survivors and working to prevent retraumatization; (5) Acknowledging the impact of child sexual abuse and institutional failures, therefore, looking out for staff wellbeing (9). This was implemented through staff training in the model, alongside ongoing clinical consultation and underpinned all work of the Inquiry.

All staff received training in TIAs on joining the Inquiry, as part of their induction. The half day training was delivered by two members of the clinical team. It included material on the neurobiology of trauma, PTSD and Complex PTSD (C-PTSD) symptoms, Dissociation, features of the TIA, and secondary and vicarious traumatisation. A further training programme was developed in on Complex Communications, which gave staff practical ways to engage with survivors via phone, email, or in person where there were complex needs and dynamics. This additional, optional training included trauma theory, impacts of abuse on interpersonal relationships, a model of abuse dynamics based on Karpman’s Drama Triangle (10), and additional material on staff wellbeing. This half day training was also delivered by two clinicians. A final training was developed, ‘Life after IICSA’ which focused on the end of the Inquiry and was delivered by members of the VSCP and a clinician. It addressed staff and survivor needs as the Inquiry drew to a close; drawing on ideas from attachment theory, models of therapeutic endings, and encouraging citizen activism as a way to reintegrate to communities post IICSA.

The programme of staff training was supported by a psychological consultation service that was offered by a range of clinicians including; psychologists, counselors, and psychotherapists. Referrals concerned various aspects of contact with survivors, including telephone contact, email correspondence, and face-to-face contact arising from attendance at Truth project sessions. There was a separate safeguarding referral service which acted in parallel. Consultation took the form of one-off or multiple meetings with a designated clinician to discuss communication, or to formulate a survivor’s needs based on their engagement. Often Inquiry staff were operating with limited information about the survivor as they were not required to provide any details about themselves, to protect confidentiality.

Staff wellbeing was also prioritized for staff across the Inquiry, originally through an employee assistance programme. However, this model evolved over the lifetime of the Inquiry, to include an additional web-based wellbeing hub, secondary and vicarious trauma workshops, reflective practice, debriefs and a compassion focused staff support group.

### Trust and epistemic injustice

Fricker’s work into *Epistemic Injustice* (1) provides a conceptualisation through which to understand the simultaneous overlaps and contestation between IICSA staff members’ roles as representatives of institutional authority and as trauma-informed, compassionate individuals. Of particular relevance here is Fricker’s clarification of testimonial justice, and the ongoing tension between viewing testimonial justice as “an intellectual or a moral virtue” (1, p. 120). Viewed as an intellectual virtue, testimonial justice is a process through which listeners (or ‘hearers’ in Fricker’s terms) are required to seek the truth of experiences regardless of prejudicial understandings of moral aspects. By contrast, if viewed as a moral virtue then testimonial justice entails the hearer valuing the wellbeing of others above the importance of the individual facts of events. Fricker concludes that testimonial justice is “a hybrid virtue” because “correcting for prejudice is necessary for avoiding missing out on truths offered by an interlocutor and necessary for avoiding doing them an injustice in their capacity as a knower” (1, p. 126).

The following subsections consider two aspects that are germane to understandings of the TIA at IICSA and also in Fricker’s work on testimonial justice: authority; and trust.

### Authority

Research conducted by IICSA into victims and survivors’ reasons for attending Truth Project sessions indicated that 50 per cent did so, at least in part, to prevent further abuse from happening (11, p. 46). Speaking their truth was a means by which victims and survivors could contribute to meaningfully changing institutional contexts and opportunities for safeguarding. A core outcome of the Inquiry’s work was indicating the scale and extent of past failures to protect children. Survivor experiences established the authority of the Inquiry to make specific recommendations to contribute toward the prevention of CSA through providing a base of evidence. At a most basic level, the sheer number of experiences shared established that CSA continues to be a matter of national concern through demonstrating the extent of the scale of sexual abuse in England and Wales, and the considerable impacts it leaves on victims and survivors. Indeed, the Inquiry concluded that CSA is “endemic within England and Wales” (12, p. 1). Through the lens of Fricker’s approach, survivors therefore contributed to the very authority of the Inquiry through providing testimonies of lived experiences.

After prevention, the next most commonly reported reason for attending Truth sessions was wanting to tell someone in authority (27 per cent) (11, p. 46).^1^ Attendees also reported wanting to be believed (17 per cent); and wanting some resolution (17 per cent). Survivors therefore emphasized the importance of Truth sessions in enabling them to share their experiences with ‘someone in authority’ (11, p. 46). Viewed through Fricker’s work (1), this might be understood as an opportunity for survivors to re-establish the epistemic validity of the speaker through participating in the formal sharing of testimony that might have been denied in prior experiences with institutions. It might also be understood as re-establishing the authority of the speaker themselves through being formally recognized by an institution as ‘telling the truth.’ These complexities of epistemic authority, truth, and trust, are all the more crucial given many survivors’ prior experiences of institutional betrayal and being disbelieved (11).

IICSA reports identified the failings across multiple institutional contexts that facilitated the widespread sexual abuse of victims and survivors. The Report of the Independent Inquiry into Child Sexual Abuse (12) explicitly identified institutional factors that negatively impacted victims and survivors, including: inadequate measures being put in place to protect children; individuals and institutions portraying children as lying; and victims being blamed for the sexual abuse (12, p. 1). While there are more specific findings into institutional contexts (13), these overarching insights indicate the lack of trust that many survivors likely feel in relation to formal institutional structures, and make clear that survivors might have ambivalent feelings about interacting with organizations.

‘Institutional betrayal’ has been found to be a considerable factor in the experience of sexual abuse in organizational settings (11). Moreover, trauma-informed literature indicates that failures by institutions to understand the needs of victims and survivors may contribute to retraumatisation (11, 14). IICSA staff were therefore given the responsibility to ensure that the survivors whose experiences they received should not be let down once more, and to limit the likelihood of further betrayal.

‘Epistemic authority’ emerges as an important lens through which to understand the layering of epistemologies and their relative authority as survivor experiences are shared and disseminated (1, p. 4). As both this and our preceding paper which addressed the experiences of survivors in the Truth Project (9) make clear, the Inquiry consistently amplified survivor experiences as the strongest form of epistemic authority: that these speakers voices conferred greater truth than others. This was a symbiotic form of collaboration in which the authority of the Inquiry was established through the collection of so many survivors’ voices, and in which the epistemic authority of survivors themselves was maintained or re-established through being listened to by ‘someone in authority’ (15).

### Trust

Trust and trustworthiness are interwoven within understandings of epistemic authority and testimonial justice, and similarly within the work of the Inquiry and the trauma-informed approach. Fricker understands epistemic trustworthiness as having two components: “competence and sincerity” (1, p. 45). From the outset, IICSA established that survivors would be allowed to speak their truth and that it would not be questioned. The Inquiry further embedded this trust in survivors through the Truth project which enabled survivors to share experiences in their own words, without established prompts or questions.

Survivors placed a great deal of trust in the Inquiry to respond to, store, and manage their data and the experiences they shared. Similarly, the Inquiry and staff trusted in the aggregated and anonymised data of survivor experiences, even if that did not take the form of specific details or the structured formatting of individual experiences. The likelihood of survivors sharing falsified experiences is very slim (16). In Fricker’s terms, this was an act of “epistemic trust” (1, p. 44) embedding the commitment for survivors to be heard and believed in their own terms and minimizing possibilities for enacting testimonial injustice.

Available data indicates that survivors of CSA describe a reduced ability to trust others (16, 17), and especially for those sexually abused in institutional contexts, a reduced trust in institutions (18). However it is important to note that recent conceptualisations within survivor research indicate it is more accurate to suggest survivors assess the trustworthiness of others and can engage in trusting relationships depending on these judgments (19). IICSA research found that 37 per cent of Truth Project participants reported that CSA “shattered their ability to trust anyone” (12, p. 78). Similarly, Palmer et al.’s research report emerging from the Australian Royal Commission into Institutional Responses to Child Sexual Abuse detailed the systemic ways in which survivors had their trust in institutions diminished through consistently being disbelieved or deemed untrustworthy (20). For survivors, this indicates the level of confidence given to the Inquiry and the immense courage in coming forward to share experiences. For staff members, this indicates a challenge in embodying both the trustworthiness of the Inquiry, but also recognizing that their own trustworthiness might come under the scrutiny of survivors.

These understandings of authority, trust, and testimonial justice provide orienting points within which to understand the role of IICSA staff members. Staff members were the medium between survivors and institutional authority: a conduit through which survivors could be ‘heard’ both in terms of their experiences valued as testimony, and in leading to societal change.

This research into staff experiences therefore aims to increase understanding of the effectiveness of trauma-informed approaches for non-specialist Inquiry staff, and also to reflect on the practice of restoring epistemic justice to those who have suffered considerable institutional betrayal on a national scale.

## Method

### Participants

Ethical approval for the study was sought via consultation with IICSA’s independent ethics research panel. Due to the study collecting evaluation outcome data, it was agreed by members of IICSA’s research ethics panel. Participants were recruited on a voluntary basis, with an advertisement for the project being shared with managers across the Inquiry for discussion within their teams. Individuals willing to participate then made direct contact with the researchers in order to find out more about the project and sign up. The purpose of the study was explained, with details of right to withdraw and confidentiality, and consent was obtained prior to participation. A total of 32 IICSA staff members participated in the interviews. Due to staff turnover it is difficult to state the proportion of IICSA staff that took part in the research study. However, at any one point there were approximately 200 members of staff, giving a suggested proportion of 16%. As well as participating in the interview, all participants were asked to complete a short, anonymous google form, recording their demographic information. A total of 26 staff members completed this form. The demographics of this group are shown in Table 1 below. The demographics of the six staff that declined to complete the google form were not recorded.

**Table 1:** Participant demographics.

Age		Gender		Ethnicity	
Over 65	0	Female	17	White/British	21
56–65	5	Male	9	Black Caribbean	1
46–55	4	Non-binary	0	Asian	2
36–45	5	Other	0	Black British	1
26–35	9	Prefer not to say	0	British Indian	1
Under 25	3				

Of the 32 participants who completed the interviews, 12 had worked for the Inquiry for more than 3 years and 14 had worked for the Inquiry for between 1 and 3 years. There were no participants who had been employed for less than a year. The participants represented teams across the Inquiry, including the legal team, communications, engagement, support and safeguarding, facilitators, operations, policy, research and facilitators. The majority of these staff were involved in the Truth Project either as all or part of their role. The exception to this were the legal team, who only had tangential contact with the Truth Project and were mostly involved in Public Hearings.

Sixteen participants indicated that they had no prior experience of using a trauma informed model and 10 reported that they did. Participants held a wide range of professional backgrounds including: civil service, social work, teaching, psychology, legal services, policy and journalism.

### Procedure

A semi-structured interview was used to gather data from the participants regarding their experiences of applying a trauma informed approach within their work for the Inquiry. This study aimed to give an expansive understanding of the ways in which training in trauma informed approaches changed how staff engaged with survivors of sexual abuse and their testimonies. As such, qualitative, semi-structured interviewing was the most appropriate approach in order not to bias participants’ responses due to researchers’ underlying assumptions, and to enable participants to develop insights that were most meaningful to them (21). These interviews were conducted by two IICSA staff members/researchers; these being a clinician (CB) and civil servant (MG).

Participants were asked to discuss their understanding of the trauma informed model, their experience of applying this and their perceptions of the responses to this of victims and survivors. They were also encouraged to give examples of where implementation of this had worked well and when it did not, as well as any impacts on their own wellbeing.

### Analysis

Qualitative data was analyzed using a six stage Thematic Analysis (22) which involved those conducting the analysis to fully familarize themselves with the data before labeling data according to the research question. Following the identification of intial themes, these were then refined and woven together in order to provide the analytic conclusions. Thematic analysis was used due to the large sample size and the qualitative nature of the data collected. This was conducted by two authors (CB & SQ). A third author cross checked coding decisions to ensure reliability across the analysis (DT). Initially, data was systematically labeled according to the research question, including line by line coding to generate initial codes. These were then reviewed to identify key patterns and define themes. Three way research supervision enhanced the reliability of the coding approach and the data was consistently used and referred back to in order to ensure credibility of those themes identified (23). Quotations are used throughout the findings in order to support the emergence of the themes identified.

## Findings*

The findings are divided into seven themes.^2^ These reflect the five TIA principles of: recognizing individual experience; fostering trust; empowerment and choice; safety and preventing re-traumatisation; and staff wellbeing. There was also a general theme relating to overall experiences of applying the TIA and how this was received by victims and survivors and a theme around personal development, including how the TIA would be applied more personally by staff members in non-professional situations. Quotes are attributed to pseudonyms to ease cross referencing and protect anonymity.

### Application of the TIA

All staff interviewed (*n* = 32) were able to describe the TIA model as developed by the Inquiry and how they apply this in their role, although with varying definitions and key words.

*“Trauma informed is being aware of the, sort of, bigger picture around trauma and the impacts of trauma umm and I think, within IICSA it’s a really positive thing that it’s so multidisciplinary. That we’ve got teams within IICSA that are not all drawn from within the civil service… We’ve got external people who come in… Um, because I think it’s important that the trauma-informed principles aren’t just principles that are written on a piece of paper and people try to follow. I think you can only follow them in a, in a meaningful way if there is that knowledge of the bigger picture, what trauma is and what the wider impact and the wide reaching impact of trauma and how that can impact on people’s interactions and sometimes behaviour, in a small number of situations, behaviour.”* (Gabriella)

In addition to discussing the key principles that are outlined below, several other principles were identified by participants including: taking a person-centered approach, listening, being non-judgemental, empathic and respectful.

*“I think the actual principles of the trauma informed approach is the way we should treat every human being anyway, is with that level of respect, you know, trying to build that trust, not treating everybody the same way, even if you think they may be the same. It’s about listening and understanding their perspective and from you know their own subjective point of view rather than just having this just blanket objective policy that you just apply to every single person in the same way, and again it’s about having those overarching principles but about being able to use your judgement and your common sense to be able to tailor them as necessary, so that you are giving that I suppose either a bespoke service or whether it’s just listening to someone, speaking to someone on the phone, over email and I think you should try and apply that in every single thing that you do, regardless of role, because they are some very basic principals that are transferable.”* (Holly)

Several participants described the overall quality of the model as having been a positive experience for victims and survivors.

*“It has been well for me it’s been michelin star for victims and survivors it’s been an absolute michelin type service they’ve been given, yeah.”* (Valarie)

*“I remember one lady saying that the police should learn from the model. Because being interviewed was horrific but had they followed the model, it would have made it so much better. So people have actually, victims and survivors that I’ve come across have actually loved the model*.” (Linda)

The importance of an approach being embedded across an organization was also identified, as being demonstrated through all staff being trained in and having knowledge of the principles.

*“Within the Inquiry, I can speak to another department or another area within the Inquiry and they will know exactly what I’m talking about when I’m talking about taking a trauma-informed approach which is a lot easier. It needs to be…in my opinion, for it to work in an organisation it needs to be… everybody needs to be doing it, not just individual departments or even individuals within an office or department doing it, otherwise it’s not effective.”* (Gary)

Further, there was also recognition that even in non-victim facing roles, such as teams that deal entirely with research and documentation, there was still an importance in all staff being trained in and understanding the TIA model.

*“I think it’s quite important to the work, so with legal I feel like a lot of the work that we do in terms of trauma and dealing with victims is more indirect so we don’t necessarily have that direct interaction with them but it really is the core and the centre of all of the work that we do.*” (Jacob)

Whilst there was a general sense that the TIA model had been effectively applied across the organization, there were some difficulties identified. These appeared to fall into two categories; training and wider organizational culture and policies.

In terms of training, individuals expressed the benefits of having a psychological consultation service which gave advice on implementing the model both within project work and in specific cases. However some participants noted that the TIA training, which occurred for each staff member during their induction to the Inquiry, should have been part of the mandatory training package that was repeated annually.

*“I think it [TIA training] should have been done after we started because yes it was helpful but I really think you need to re visit and revisit not as just training but small group discussions so that its stuck for life so it reinforces what we’re doing, the practise that you’re getting right and it gives you ideas, that’s what you need- ideas how when you’re in the interview you can make the experience as good as it can be and promote trustworthiness, empowerment, safety you know how you can come out things from a cultural agenda perspective, we need to constantly constantly revisit.”* (Shannon)

There was also a concern that, whilst trained in and given additional advice when needed, there was no monitoring or feedback with regards to implementation of the TIA model or how individuals could further develop their skills.

*“I’ve never had for myself the equivalent of an observed lesson. I’ve never had anybody say to me, I was listening to the tape of that session, this was good, that could have been better, you know, you talked a bit too much here, whatever. Or have a conversation about it, the only time I’ve ever had any feedback, I mean yes we do the closed session debrief and I think that's valuable, that’s not a situation for that to happen and apart from anything else there is the power relationship, you know, the (Truth Project) facilitators are unquestionably I’m afraid, hierarchically above assistant facilitators, I don’t think it’s right but it’s a fact and so that's not a position where assistant facilitators will feel empowered to give a properly objective view of what the (Truth) session was like, so always you talk over practical things and stuff but then if you try and ask assistant facilitators how they think it’s gone it will be all, you know, “oh well I think it’s good”. It’s nothing really.”* (Robert)

With regards to organizational culture, the Inquiry was independent of government but sponsored by a government department and employing civil servants. As a result, it was noted that there was sometimes conflict between the target driven culture of needing to get the job done, and the more trauma-informed perspective of needing to be flexible and adapt to individual needs.

*“I think it [civil service] can be a barrier because I think the approach is, if i was to say it’s a very civil service approach I think that there’s a lot of things that are packed up in that…Overly hierarchical, complex bureaucracy, buck passing so an inability to take decisions because there’s a concern about if you take the decision and it’s the wrong decision, a focus upon process above substance sometimes, that would be what I would say are the main barriers because it’s all quite process driven rather than “how do we get the job done?”*” (Vera)

There were some reports that this resulted in increased complexity of tasks that might otherwise have appeared to have been straightforward.

*“I think sometimes it can…I think too many people can be involved sometimes. If you send an email, erm – one experience that I had, I sent an email and there ended up being a chain of about…36 emails with one case, with over 10 people involved. And by the time I’d got to the bottom of the emails and worked out what was happening and everything, I was completely and utterly lost.”* (Sarah)

### Recognizing individual experience

A total of 37.5% (*n* = 12) of participants made specific reference to the need to recognize individual experience and to treat everyone as an individual when asked to identify the key principles of the TIA.

Feedback from some staff appeared to reflect how their work within the TIA model resulted in enhancing their knowledge of individual differences and how victims and survivors all react and respond to trauma differently.

*“It’s definitely made me understand that everyone thinks and feels differently which is why I think the trauma informed approach is in place everyone has a similar guideline and a similar approach to follow in the inquiry but it’s definitely made me more considerate of how different things can make people react differently so what might trigger me might trigger someone else or what might be stressful to me might be stressful to someone else and so I think the trauma informed approach has helped me understand that.” (George)*

This increased understanding seems to have really challenged individual expectations about what the response to a trauma should or does look like.

“*There cannot ever be an objective measure of personal trauma. You know I’ve had participants who I think have been more powerfully affected by what you might think looking objectively from a distance to have been really quite a small thing than others who have experienced something which objectively looking from a distance people would say “oh yeah, that’s proper trauma that is” so I don’t think you can ever really judge “how traumatised has somebody been?”, all that matters is what the effect has been on them. What their experience of it is.”* (Robert)

For others, there was a sense that perhaps, in enhancing a TIA approach, we expect victims and survivors to be more vulnerable and to perhaps present as having more difficulties than, in reality, many do.

*“I do think we, with the best of intentions, maybe lose a little bit of sight of the fact that our participants are resilient, capable people who are living their lives and are making a choice to come to us. I think sometimes we can become a little bit, um overly solicitous and I think, when you look at some of the feedback, you know, that we see through victim and survivor studies, we know that one of the many reasons that people don’t speak and don’t come forward is because of a fear of how they will then be perceived. It’s a fear of that victim status being attached to them and a fear of them being seen as less capable, less strong and less resilient when actually the opposite is true and I think we know that and we say it but then, with the best of intentions, sometimes we become overly solicitous which could, sort of, reinforce those fears that in some participants.*” (Gabriella)

### Fostering trust

In total, 37.5% (*n* = 12) of participants identified trust as being important when asked to identify the key principles of a TIA.

A key ingredient in fostering trust that was identified by several participants was transparency and that, in order to build trust with victims and survivors, Inquiry staff needed to be transparent about what they were doing and why.

*“It’s about being completely clear and transparent with them…otherwise our decisions would seem completely arbitrary and we’re just making a decision because we feel that we should where as at least I can, I always refer to the protocol and I always provide a link to the protocol so that they can see for themselves why we have applied the redactions that we have applied. So yeah as I say, being completely transparent, being concise and being clear to them so that they can understand for themselves why we have done what we’ve done.”* (Jacob)

There was also a recognition that, at times, being transparent means being open about things that cannot be done or questions that cannot be answered, rather than generating a false sense of hope.

*“The trauma informed approach is to make sure that we’re not making false promises as well, that we’re as realistic as possible and it’s about managing those expectations and sometimes we have to deliver bad news, your support has ended for example, we can signpost you, that’s as far as we can go. Obviously it has to sit within the inquiry’s remit as well, so we can't help everyone and I think it's about recognising that as well.”* (Holly)

Alongside this openness, there was a recognition of the weight of responsibility on staff as individuals to follow through with what they say they will do, so as to not let victims and survivors down.

*“I know I’m comfortable enough to say to a victim and survivor listen I can’t answer that question or I’ll get back to you and I’ll seek advice and get back to them and again one thing is for that is that we do, when we say that we get back to people, we get back to people.*” (Andrew)

Similarly to Paper One, the biggest reported barrier to trust was the Inquiry’s responsibility to report allegations to the police. This was identified as a key difficulty in both building trust and a trigger for trust breaking down.

*“… as soon as you bring up the police that does seem to be a trigger point for when a lot of people will just choose to disengage… maybe if I didn’t bring in the police right then or if I’d kind of led into it a bit more, kind of go into this section discussing around police involvement…A lot of the time it is having a conversation around taking details for a session. And then you’re asking a serious question around police consent. And in their mind that means that an officer’s going to be knocking on my door. So. And we can’t say that’s not going to be the case because it might. Even if we say, “please don’t go and knock on their door, email them first”, they might just go round and knock on the door, so…yeah. And that’s…feeling that – do you want to do…making promises or assurances which you can’t guarantee with 100% certainty.*” (Harry)

However, it was acknowledged that, even with knowing the potential impact of the role of the police, this information still needed to be discussed openly with victims and survivors.

*“We have to give them the ownership of what they want to do. So quite often will just explain the remit of the inquiry, if they want to take part in the Truth Project, what that entails. We also inform that we will have to tell the police if they give us any information about abuse, but that will also be done anonymously so that they don’t have to give their contact details, cause a lot of people do worry about that. They may of had a bad experience with the police, obviously we have to tell them, we have to be open and honest and tell them what we’ll do with the information that they give us.*” (Laura)

### Empowerment and choice

In total, 15.6% (*n* = 5) of participants referred to empowerment and the need to offer choice when asked to identify the key principles of a TIA. Below are details about environmental and comfort that were also identified as helpful by survivors in the first paper.

*“We try to make it as pleasant as possible for them, so we give them the choice of setting out the room and anything that might make them feel comfortable, biscuits, tea, flowers, whatever, just to make it feel more homely, and their in control, I think thats the main thing, that the victim and survivor is in control of everything, were not controlling it, their the ones in control, their helping us, were not interviewing them or interrogating them or whatever, so I think from that perspective.”* (Laura)

There was a considerable overlap between the principles of trust, empowerment and recognizing the needs of the individual. In particular, in acknowledging that different individuals will make different choices and that it is not for Inquiry staff to override their individual choices.

*“It’s their decision and their choice what they share, how they share, if they share. It’s their decision if they complete a session or not. It’s their decision if they want a break just to recharge, regroup and come again. It’s totally their decision and it has to be about their decision. We’re just, I don’t want to say a bystander, we’re just there to facilitate the journey. We’re not there to direct the journey. So for me, it has to be about their choice, it can’t be any other way.”* (Valerie)

However, there was also a rationale for not offering too much choice and having boundaries within what is available, with boundaries being seen as positive containment as opposed to being restrictive.

*“We want to give our participants as much choice as possible and to meet every need that we can possibly meet. But sometimes there’s empowerment in setting an appropriately and sensitively set boundary. Um, because I also think you don’t do people any favours when you behave in a way that gives the impression that there are no boundaries on your interaction with us.”* (Gabriella)

### Safety and preventing re-traumatisation

In total, 56% (*n* = 18) of participants identified safety and the need to avoid retraumatisation when asked to identify some of the key principles of a TIA.

It was clear that avoiding retraumatisation was a core value in many staff, and that people had a real motivation to help victims and survivors cope well with their experience of engaging with the Inquiry.

*“The legal team that I work with are all quite sensitive, switched on women and we’re not just robots and just got through the process to get the witness to give evidence and you know, in and out the witness box and thanks very much off you go. We want people to feel positively about their engagement with us as an Inquiry but also us as individuals.”* (Hannah)

Many staff members were able to recognize signs of distress and offer examples of steps taken in attempts to create a safe environment in Truth sessions in which victims and survivors could share their experiences.

“*…one woman who was in the chair, she was physically shaking. She was, she didn’t know what to do with herself, she was fidgeting and she was shaking, she was literally… she wasn’t at ease at all. So what I, what I did was spent a little time explaining how it was gonna work and explaining that it is about them and if they need to stop, well I give people, I say to them at the beginning, look you tell me as much or as little as you like, at any point, you change your mind, anything like that about the recording, at any point do you want a break, just have a cup of tea or go out and get a bit of fresh air or have a cigarette if they smoke, you’re welcome to do that. And if it’s too much and you want to leave, you can do that, you don’t have to stay. You don’t have to be here to tell me because it’s about what you can cope with and just, just making sure that’s alright and just check that they’re okay. You know, check that, you know, if there’s anything they need, do they need, if they’re getting upset, do they need a break. And I think by the end of it, she had, she stopped shaking, she was calm and she was really comfortable.”* (Valerie)

Some staff recognized that, as difficult as sharing was, for some individuals this was a cathartic process and working through sharing helped with healing.

*“The key thing is being aware of the long term pervasive and not necessarily obvious effects of trauma on an individual and so trying to bear those in mind to ensure that we don’t re-traumatise but that also we kind of provide opportunities for somebody who has suffered trauma to communicate effectively to heal this and obviously in an ideal world to have some sense of having been able to do something about their trauma by kind of giving witness… The sense I get is that people find the whole process much less intimidating and kind of traumatising than they expected it to and quite often, people explicitly say and if they don’t say it you get a very strong sense of it, of the kind of “handing over a burden”* (Robert)

However, there was an acknowledgement that, whilst the approach may have avoided re-traumatisation for many, it could not avoid it for all.

*“I have one person…who after ten minutes could not go on, unable to go on because he was retraumatised. It was the first time he had ever shared the story and he thought the would be okay but he wasn’t so when we did the session within ten or 15 minutes but that’s the only example of that kind.”* (Shannon)

*“There was a lady who, it was so traumatic for her, she started having chest pains. She was having chest pains so I actually had to stop the session, so I stopped it and just said to her, look you’re obviously in distress, let’s take a break. And she said, yes that would be good. She went to the toilet and she came back and she was still having chest pains so I actually said to her, look what we will do, let’s stop the session, we can re-book and continue if you wish to do so, and if you don’t want to come back, that’s okay but for now, I don’t want to put you through anymore trauma than you’re going through because physical chest pains rings alarm bells in my head, a. because she’s struggling but there’s also a health implication there as well. So, I did stop and she did re-book and she did come back.”* (Linda)

In this case, while the Truth participant showed signs of a strong physical reaction that might have been a precursor to a retraumatising experience, with the offer of choice and a relational approach from the facilitator, she was able to come back and complete her testimony. This is reminiscent of the finding in Paper one that suggested for some survivors, difficult experiences in telling their story should not be assumed to be retraumatising.

### Staff wellbeing

In total, 18.8% (*n* = 6) of participants referred to staff wellbeing as a key principle within a TIA.

The importance of staff wellbeing was clearly identified by staff members across a variety of teams, focusing upon the impact of this upon their engagement with victims and survivors.

*“I think that there is a culture of care because that’s you know that’s about looking after those of us who work because if we’re not looking after ourselves or we’re not being looked after we can’t offer a proper service to a victim and survivor coming through the door.”* (Gemma)

Overall, most staff reported being aware of the various sources of support across the Inquiry and were able to identify where they would go for support if they needed it.

*“I certainly feel the kind of like supported and protected in the information that I come across and what to do if it’s kind of too much you know.”* (Lily)

There were also a number of strategies identified for managing those situations that staff may find triggering.

*“I think what I’ve learnt to do now myself is that if I’m reading a document and it is particularly sort of it hits close to home or something I think I will allow myself the time to take 10 minutes away from my work… get a fresh air or get a cup of tea and just let my line manager as well that I’m going to be doing this and I know they are very supportive of it but it was not something I was necessarily aware of that I could do at the beginning and it was something I had to figure out for myself. But I think yeah they are, everyone is really understanding but it’s more of a case of just you have to be more vocal of it from the beginning just so that I’m aware that I can do that if something is particularly difficult then it’s the case to sort of take a break.”* (Shana)

However, there were some teams where individuals reported that measures to support staff wellbeing did not appear as evident.

*“When I was working in the legal team…and I would listen to victims and survivors giving evidence or I’d have to deal with them behind the scenes and that was obviously very emotionally charged and very distressing. At the time there weren’t any measures in place to ensure, the (clinical) team were there but they were there predominantly for the witnesses I don’t think there was anything there for the legal team or the people who are behind the scenes making the hearing happen so I think in future that would be something that would be good to have because I don’t think I thought about it until, thinking about it now retrospectively I’d come home and I’d be quite, I wouldn’t say miserable I would just be quite deflated from the day, not having a chance to debrief.”* (Alice)

These difficulties appeared to be particularly heightened when there was a pressure upon staff in relation to tight deadlines and the need for tasks to be completed quickly.

*“When I worked as a (member of the legal team) I found it very difficult because the emphasis was on redacting a large volume of material and getting a lot of documents disclosed to core participants in a very short period of time, so the emphasis was very much on quantity, producing a lot of material and redacting a lot of material and I think there wasn’t a lot of emphasis in ensuring that we’re taking a trauma informed approach.”* (Alice)

Staff wellbeing was also dependent upon the team in which someone worked, with recognition of strategies that were used across the Inquiry, but that the specific team approach or management style would impact upon how supported individuals actually felt.

*“I’m not as confident in how well the Inquiry and we as a team, as a wider team and also as a research team work within a trauma informed approach with each other. I feel like we’ve got the structures, we’ve got like the framework, of you know, the wellbeing checks…and we’ve got mental health champions so it's kind of there but I feel it’s almost, bear with me with my analogy, it’s almost like the framework you know when you’re putting a gazebo up and you’ve got your frame up but you haven’t got the cover on yet and I feel there’s a little bit of that with it in terms of staff. And I’ve thought about it a lot because I’ve had to, I’ve had some struggled with my responses to some of the content because I’ve had a period of being very immersed in Truth data and what I have found is, and obviously this is just my very personal experience now…I haven’t really known where to go with that and yes, there is someone saying we’ve got (wellbeing service) and Im saying “well, I’m not sure about (wellbeing service) because I’ve done the wellbeing check which was ok, but it wasn’t wasn’t that useful”…I don’t really want to un… to delve right down into it again with some counselling, I think that’s very destabilising, I want someone, I need a pathway to help me that’s very private and very confidential and puts me, my little bit of it in the centre to help me do my work and actually what’s happening is I am trying to manage it on my own and some days are ok and some days aren’t.”* (Maria)

There was also a suggestion that accessing support should be a more formal requirement to ensure that staff wellbeing was prioritized.

*“Before this job I worked at (another Inquiry) so I was and I think that that framework for how they looked after their staff, I thought was absolutely incredible and a few differences to (IICSA), the only thing I would say is I think is the, I would say it probably was trauma informed, the (other Inquiry) was more proactive on the support aspect for employees so we had a one hour consultation per month with a support worker which was mandatory and, not mandatory but highly encouraged most people took that up including myself and it wasn’t waiting for someone to hit a point where they felt they needed to have to reach out… it was kind of set up and part and parcel of kind of the job that we did to make sure we had that hour to talk about, the same as (IICSA wellbeing service) it could be anything to do with your job or something else or secondary trauma, things like that so I have I would say yes I have before I think.”* (Elizabeth)

### Personal development

As well as feeling that involvement with the Inquiry had a positive impact upon victims and survivors, many staff identified the changes they had observed in themselves as a result of their work.

*“It has its made me a more understanding person, someone who listens more and considers other people and also thinks ahead.”* (George)

There was also an identification that individuals cannot be involved in this work and the boundary between the professional and the person become less clear, given the level of emotion involved. This was perceived as something beneficial that is relevant to future areas of work.

*“I think something quite generally that I have found really helpful about the approach is how it encourages you to integrate professional and more human stuff, which I think sometimes in the workplace, I was working in (another country) before where there is a really strict line between professional and personal. So I find it really helpful for there to be an institutional integration of those things; we are not a robot doing work, we are human and that means X Y Z and kind of formally recognising those things and integrating them into the institution’s work is so positive. That’s definitely something I would personally take forward; making sure that I’m a policy adviser first but also a human too. Wearing two hats I think.”* (Rachel)

For some staff, there was a recognition that their work within the Inquiry had changed them and their approach to thinking about CSA, often resulting in others being more open with them about their experiences and the staff members being able to offer more support. This was spoken about in positive terms as opposed to being a burden of the work.

*“I think before I started on the Inquiry, no one spoke about it, but I think since working here, obviously people know what I do to a certain extent, everyone seems to be talking about it which I think is good. I think it’s good and that's probably, hopefully what we’re aiming for it to be more open, and obviously places where children can go and tell people about it so it’s not so hidden. So I think it’s definitely a good thing and I think working here has made a difference wider world…. I’m very surprised really, might be unusual but I would say at least ten people have disclosed to me that they were abused as a child, which obviously is not what you want to hear from your friends, but then again I think it's changed me as well because I’ve been supportive. I’ve not been talking in great depth about it, but just for them to tell me I think is probably a big thing for them.”* (Laura)

Whilst disclosures were perhaps an unexpected consequence of working within the Inquiry, the knowledge and training relating to a TIA appears to have better equipped staff for managing these situations in non-professional contexts.

*“I feel like it’s sort of made me aware of the sort of um, how common, how common it is and how it impacts sort of sadly the majority of the population in some way or another and I think from that perspective it has given me the ability to sort of deal with it so for example I have, a lot of my friends are obviously aware of my job and the work I do and they have felt since me, I’ve only been here for a year, but since me being here a lot of my friends have come forward to me about their experiences and I just feel equipped to be able to sort of signpost them in the right direction but also just sort of taking that time to be sort of understanding to them and supportive to them and so I think that has really helped.”* (Shana)

## Discussion

This research indicates that IICSA staff could clearly identify TIAs and all could identify the core elements of these approaches, suggesting a baseline training was helpful in creating a shared orientation. There were interestingly diverse views on the relative vulnerability of different survivors, suggesting for some staff there was an overcaution around vulnerability that may have missed underlying forms of resilence. This fits with findings from survivor experiences in Paper One. More than half identified the importance of survivors’ safety in sharing experiences with the Inquiry, with contact with the Police being a significant trigger point for many survivors, a finding that also corresponds with survivor perspectives in Paper One.

The qualitative data indicates the prioritization of survivor wellbeing when engaging with the Inquiry and the Truth Project. Staff described dealing with highly distressed survivors who at times displayed concerning psychological and physical reactions to the stress of talking about CSA. The descriptions of staff responses are sensible and appear to follow a TIA approach, suggesting that both the staff training and ongoing consultation provided was helpful in at least some cases in creating safety and responding to risk. Given that the majority of staff in this study were not clinicians, this is a considerable finding. Similarly to the survivor experiences, a more nuanced view of retraumatisation emerges, suggesting for some survivors reliving their distress in a truth session was not inevitably destructive in achieving their aim of providing testimony.

The qualitative data also indicates a more complicated landscape in relation to staff wellbeing, particularly within an outputs-oriented context such as the Inquiry. As in the survivor experiences in Paper One, there was a small but important minority who struggled with the CSA material in a way that indicates some negative impact. At the same time, many staff recognized positive changes in relation to personal development and changes in their relationships to others.

The findings will now be considered through a Epistemic Justice lens, followed by consideration of their implications for the TIA literature. Strengths and limitations of the study will also be discussed.

### Testimonial sensibility

One aspect that emerged strongly in the data of IICSA staff experiences of TIAs is the curation of testimonial sensibility. Fricker conceptualizes testimonial sensibility as: “where a hearer gives a suitably critical reception to an interlocutor’s word without making any inference” (1, p. 71). In the Inquiry’s understanding, this ‘critical reception’ entailed a commitment to recognizing survivor testimonies as truthful and sincere, and without requiring challenge or clarification in order to verify. IICSA staff orientation to the credibility of survivors in some cases led friends to reveal their own experiences outside of the workplace.

Staff members consistently referred to the importance of receiving survivors’ experiences in their own terms, without being guided or compelled by IICSA staff. Fricker’s concept of “neutrali[s]ing prejudice in credibility judgements” is relevant to understanding this orientation of staff in relation to survivor testimonies, as well as testimonial justice (1, p. 122). ‘Neutralising prejudice’ involves removing judgments regarding the credibility of speakers in order to enable testimonial justice. While Paper One developed an understanding of the impacts of debates surrounding False Memories, the experiences of IICSA staff reveal the challenge of being the hearer of experiences we know to be true, and with which we would like to effect social change.

The experiences of IICSA staff reveal the complexities of requiring individuals to suspend or internalize emotional responses in order to present neutralized engagement. To be clear: the challenge was not a case of survivors not being believed, but rather that neutralizing credibility judgments entailed limiting the moral and emotional judgments of hearers in order to limit testimonial injustice. In this sense, testimonial injustice might emerge through an excess of moral and emotional engagement by the hearer. Receiving, synthesizing, and presenting the ‘truth’ of the considerable institutional failures to protect children from sexual abuse required staff to manage emotional responses to challenging material in order that emerging data might not be deemed prejudiced by individual sentiment.

However, individual staff members could not offer indications or guarantees to participants that there would be any specific social and political changes, nor any concrete actions taken on the basis of experiences shared. Moreover, facilitators were required to inform participants that any indication of ongoing criminal acts would be immediately handed to the police. Staff members thus occupied an interstitial zone between institutional authority and individual engagement that might raise considerable challenges. While testimonial justice is certainly a ‘hybrid’ of both moral and intellectual virtues, this very hybridity recognizes the competing tensions of impartiality and affective engagement embodied by IICSA staff.

Staff working at IICSA recognized the authority they had in relation to survivors and survivors’ data. ‘Authority’ here captures multiple meanings. While explicit that sharing an experience with the Inquiry did not necessarily entail formal recognition of the individual facts of people’s experiences, nonetheless sharing an experience with the Inquiry conveyed a sense to survivors that their experiences were being listened to by those in positions of power. This sense of formal authority became embodied by individual staff members in Truth sessions. At the same time, those working in other areas of the Inquiry enacted authority over the representation of survivors’ experiences and management of survivors’ data.

This framed IICSA staff members as representative of authority structures which might include both institutional opportunities for restitution, redress, and reform, but might also include coming to represent the institutional and organizational structures that initially facilitated the sexual abuse and failed the survivor. This challenge for staff of both representing organizational structures that failed to protect children from sexual abuse, and as embodying the possibilities for institutional reform captures the epistemic challenges of receiving and working with survivor testimonies.

As no individual staff member held authority over every aspect of the Inquiry, IICSA staff also placed their trust in ‘the Inquiry’ body overall to produce the most truthful and impactful findings and recommendations emerging from the collected testimonies and data. Located between the empathetic and engaged relationship to survivors and survivor data and the structural requirements to process and present findings as dispassionately as possible, staff members had to trust both that the testimonies they were given were truthful, and that ‘the Inquiry’ would produce meaningful change.

At the same time, individual survivors might view facilitators as enacting forms of epistemic authority over their experiences and consider that IICSA staff were seekers of dispassionate truths and might listen to their experiences with prejudicial skepticism. IICSA staff might also feel the responsibility associated with being in a position of epistemic authority, and conscious that the capacity to effect social change relied on their ability to effectively represent the experiences of victims. These complementary and competing formations of epistemic authority point toward the layering of ‘trust’ between survivors, staff members, and the Inquiry more broadly.

### Trust

One of the most powerful themes to emerge in the data relates to the trust placed in staff, and the Inquiry more broadly, by survivors. This trust included the ways in which the Inquiry collected, stored, and deleted people’s data, but also included trust that staff would try to diminish possibilities of retraumatisation. Staff members recognized the trust placed in them as individual representatives of the Inquiry, and evidently felt a responsibility to ensure that they maintained this trust.

A slightly more challenging area that the data points toward is the tension between the empathetic listening and support that constitutes important elements of TIAs and where survivors disclosed information which would be necessary to disclose to police. While staff understood why there was the need to contact the police if details were disclosed indicating that someone might be at risk of harm, they also understood that this mandate could deter survivors from engaging and might inflect or jeopardize the ways in which they related to the Inquiry. While further research would be needed here, available research indicates that many survivors of sexual abuse have negative experiences with the police and the criminal justice system more broadly (24). Moreover, telling survivors that their details might be shared with the police made manifest the power differentials between survivors and Inquiry and Inquiry staff which might otherwise be diminished through TIAs.

While the TIA worked to diminish the harm to survivors in engaging with IICSA, nonetheless the tensions between the institutional authority of the Inquiry and individual survivor vulnerabilities could emerge. This arose in the qualitative data in staff relating the importance of setting boundaries and limiting expectations for participants, which metonymically indicates an impermeable limit between survivor engagement and institutional responsibility. This sense also emerged in staff talking of survivors receiving a “michelin star” and “bespoke” service which indicates a transactional rather than relational element to participants’ sharing experiences. This sits at odds with Fricker’s conceptualisation of a Testimonial Sensibility, which emphasizes the “idea that our responses to others are learned and internalized through a process of epistemic socialization: a social training of the interpretive and affective attitudes in play when we are told things by other people.” (25, p. 161). While survivors might receive an exceptional service, the transactional wording also recognizes that IICSA staff received salaries to conduct the work. It also gestures toward the limitations of institutional engagement, such that survivors might experience the outward presentation (similar to diners in a restaurant) while not being allowed to see the preparation or perceptions of the staff and institutional structures.

On the other hand, this process of ‘epistemic socialization’ was present for staff in the implementation of the TIA as evidenced by the shared understanding of core principles such as offering choice, safety and empowerment. The extent to which this was generalized for some staff to external roles can be seen in the broader shifts in attitudes to CSA their contact with survivors elicited.

### Policy and practice implications for TIA implementation in inquiries and other settings

One of the novel features of this study is that staff trained in the TIA were not in the main experienced in working with survivors of CSA, and so had no pre-existing paradigm that had to be challenged. This is in contrast to many staff working in mental health settings where TIAs represent a shift from medicalised approaches where trauma is backgrounded (25). The lack of ambivalence about the TIA implementation here contrasts with other studies (8), suggesting that concerns about TIA training for non-clinical staff may be misplaced as they can come to the material unencumbered by competing allegiances. This finding validates other studies which also found evidence to support non-clinical staff training (5, 6).

A second practical implication is that the concerns about exposing staff to trauma related materials central to CSA appear to be overemphasized, but not absent, in the context of TIA implementation. For most staff interviewed, the impact on their own wellbeing was limited however for those who did struggle, additional support was needed. The suggestion that the wellbeing offer be made mandatory rather than optional is interesting, and links to the challenges of recognizing early signs of burnout, or vicarious and secondary trauma in oneself and others.

While CSA remains a highly psychologically disturbing and stigmatized area of social life, as IICSA itself found (26), there is encouraging evidence in this study to suggest, if well supported, staff can tolerate distressing CSA material and support survivors appropriately. This aligns with child abuse inquiry scholarship which has suggested that Inquiries serve an important function in spearheading wider societal recognition of child abuse (27), and can create new discourses that privilege survivor accounts over institutional expertise, the ‘turn to testimony’ (28). From this vantage point, IICSA staff might be considered a microcosm of evolving societal responses to victims of child abuse, suggesting new forms of validation and respect that have important dignity conferring functions for survivors (29). Caution should be employed however in overstating the value of a purely therapeutic sensibility in the absence of wider justice considerations (30). In addition the highly controlled and well resourced context of IICSA and the Truth Project may not be easily replicable in other environments, where survivors continue to face stigma and prejudicial treatment.

One broader policy implication for future Inquiries is that the training offered here was fairly brief and yet in combination with other forms of support, has had a substantial impact on staff practice and the wider environmental milieu. This means that future Inquiries into areas other areas of challenging social areas that require engagement with impacted citizens, can considerably enhance effectiveness in engagement and also attend to staff wellbeing by implementing a similar model of training and support.

## Limitations of study and future research

Similarly to the Truth Project Paper One, this study was conducted by IICSA staff, with a risk of bias inherent. This was somewhat mitigated by the methodology of triangulating analysis via two researchers and a supervisor. What the insider status offered was access to the whole staff group and institutional support which may not have been possible for independent researchers.

The qualitative approach in this research study enabled participants to provide granular and reflexive insights into a topic that can be challenging and emotional. Complementarily, although pointing to wider learning on trauma informed approaches, the findings of this study are limited to the participants of this research. As such, they are not necessarily representative of wider populations, nor would it be possible to reproduce the study to test the validity of its findings. Lacking human resource data, we are unsure whether the participant characteristics of the sample are representative of the wider staff characteristics at the Inquiry. While there were standard themes asked of all research participants, the semi-structured approach to interviewing meant that questions and responses were not standardized. These limitations severely curtail the generalisability of the findings and the possibility of conducting rigorous quantitative analysis. Nonetheless, the depth of the insights provided by participants constitutes its own form of data validity that present difficulties with larger cohorts and withe more formal research approaches.

This study found much to recommend Fricker’s work on Epistemic Justice as applied to the treatment of the testimony of survivors of CSA in Non-recent Child Abuse Inquiries and other contexts. One possibility is the use of the key areas outlined here- the promotion of testimonial justice through attention to an ethics of listening, neutralizing credibility judgments, and managing allegiance conflict for staff- could be implemented into TIA training and evaluation, which has been critiqued for a lack of operational and conceptual specificity (31).

## Conclusion

This study found encouraging signs that non-specialist staff can be trained in TIAs and are able to work sensitively and safely with child sexual abuse survivors in a non-recent child sexual abuse Inquiry setting.

There was some concern about the impact of CSA material on staff wellbeing but less than is popularly presented as a barrier to working with trauma. There were a number of overlaps in findings with staff and Truth Project participants around a nuanced approach to retraumatisation, the interference of outside institutional influence on TIA effectiveness, and the centrality of relational factors in all aspects of communications. There was support for the application of Epistemic Justice in considering staff working with CSA survivors, particularly around the need for a testimonial sensibility that pays attention to the complexities around different forms of authority and the communication of a non-prejudicial listening that compensates for historic injustices in this field.

## Data availability statement

The raw data supporting the conclusions of this article will be made available by the authors, without undue reservation.

## Ethics statement

The studies involving human participants were reviewed and approved by IICSA Independent Ethics Committee. The patients/participants provided their written informed consent to participate in this study.

## Author contributions

CB: principal investigator. DT: research supervisor and co-wrote manuscript. MG: project management and data collection. SQ and SF: data analysis and write up. RE: project oversight and organizational leadership. WT: co-wrote manuscript. All authors contributed to the article and approved the submitted version.

## Funding

This work was supported by the Home Office UK.

## Conflict of interest

The authors declare that the research was conducted in the absence of any commercial or financial relationships that could be construed as a potential conflict of interest.

## Publisher’s note

All claims expressed in this article are solely those of the authors and do not necessarily represent those of their affiliated organizations, or those of the publisher, the editors and the reviewers. Any product that may be evaluated in this article, or claim that may be made by its manufacturer, is not guaranteed or endorsed by the publisher.

## Footnotes

^1^This excludes ‘Other’ categories in which participants could provide reasons not listed in the ‘reasons for attending.’

^2^The research findings coming from this study do not constitute formal recommendations by the Inquiry’s Chair and Panel, and are separate from evidence obtained in investigations and hearings.
